# Resistome Profiling of a Large Collection of *Staphylococcus aureus* Isolates Uncovers Frameshift-Silenced *mupA* Gene Mediating Mupirocin Susceptibility

**DOI:** 10.3390/ijms27093764

**Published:** 2026-04-23

**Authors:** Martyna Kasela, Katarzyna Suśniak, Mateusz Ossowski, Anna Malm

**Affiliations:** 1 Department of Pharmaceutical Microbiology, Medical University of Lublin, 1 Chodźki St., 20–093 Lublin, Poland; martyna.kasela@umlub.edu.pl (M.K.); katarzyna.susniak@umlub.edu.pl (K.S.); 2 Department of Animal Hygiene and Environmental Hazards, Faculty of Animal Sciences and Bioeconomy, University of Life Science in Lublin, 13 Akademicka St., 20–950 Lublin, Poland; mateusz.ossowski@up.edu.pl

**Keywords:** *Staphylococcus aureus*, antimicrobial resistance, infection, colonization, silent resistance genes, mupirocin

## Abstract

*Staphylococcus aureus* is a high-priority pathogen causing skin and soft tissue infections (SSTIs). The frequent resistance to anti-staphylococcal agents exhibited by this underscores the need for accurate diagnostics to guide effective therapy. Therefore, this study aimed to compare phenotypic and genotypic resistance in *S. aureus* isolates from nasal carriers and SSTIs and to elucidate gene-silencing mechanisms. In total, 355 *S. aureus* isolates (256 isolated from carriers and 79 from SSTIs) were studied for their phenotypic and genotypic resistance to β-lactams, macrolides, tetracyclines, aminoglycosides, and mupirocin. The silenced *mupA* gene (low prevalence: 0.6%; 2/335), linked to mupirocin resistance, was sequenced, and expression was assessed via reverse transcription qualitative PCR (RT-qPCR) in all *mupA*-positive isolates. SSTI isolates showed significantly higher resistance to erythromycin, gentamicin, and mupirocin, along with a higher prevalence of multidrug-resistant strains and *ermC* and *tetM* genes. Sequencing revealed multiple mutations in silent *mupA*, including a critical frameshift (c.372 delA) in a poly(A) tract that brings about premature truncation. RT-qPCR indicated upregulation of silent *mupA* variants and high variability in functional strains, suggesting that frameshift alone prevents resistance. These findings highlight silent resistance genes as key targets for advancing *S. aureus* surveillance and for combating emerging threats.

## 1. Introduction

*Staphylococcus aureus* is a bacterial species widespread in the environment and a common human pathogen causing both community- and hospital-acquired infections worldwide. It ranks as a leading cause of skin and soft tissue infections (SSTIs), which range from mild to life-threatening and may be associated with high mortality [1,2]. This pathogenicity is closely tied to *S. aureus* capacity for asymptomatic carriage, particularly in the nasal cavity, which creates a reservoir for endogenous infections at other body sites [3]. Approximately 20–30% of the general population harbors persistent nasal colonization [4], with pooled European data from nine countries estimating a prevalence of 21.6% (95% CI: 12.7–29.4%) [5].

Compounding this public health challenge, *S. aureus* has developed resistance to multiple antibiotics. The rise of methicillin-resistant *S. aureus* (MRSA), alongside resistance to common anti-staphylococcal agents like macrolides, clindamycin, tetracyclines, and co-trimoxazole, has driven the emergence of multidrug-resistant (MDR) strains [6]. Consequently, the World Health Organization (WHO) classifies MRSA as a high-priority pathogen, posing a major global clinical threat [7]. Therefore, addressing diagnostic issues, such as discrepancies between phenotypic and genotypic results, is crucial for implementing effective antistaphylococcal treatment. Routine microbiological diagnostics in most cases rely on assessing phenotypic antimicrobial resistance, which, for obvious reasons, leads to a bias toward resistant strains. Even in scientific studies investigating genetic determinants of antimicrobial resistance, the focus is typically on the subset of strains exhibiting phenotypic resistance. As a result, susceptible strains harboring silent or inducible resistance genes—due to mutations like frameshifts or reliance on stressors (e.g., antibiotics during therapy, oxidative stress)—are often overlooked and remain unrecognized until activated.

In general, there are two possible scenarios explaining the discrepancy between genotypes and phenotypes in bacterial isolates in terms of antimicrobial resistance, i.e., when an isolate demonstrates phenotypic susceptibility in vitro while harboring genes responsible for resistance. The more prevalent scenario occurs when induction (exposure of bacteria to an antibiotic in their environment) is required to activate a resistance gene and confer phenotypic resistance, often in combination with additional genes or regulators. This phenomenon is well described in *Staphylococcus* spp. and most often involves the *blaZ*, *mecA*, and *ermA* genes, conferring resistance to penicillins, β-lactams, and macrolides, respectively [8].

The second scenario involves the presence of a nonfunctional gene due to various genetic defects, most commonly mutations. This phenomenon is referred to as “silencing of antibiotic resistance by mutation” (SARM), a term first introduced in large-scale studies by Kime et al. [8]. In such cases, the gene is considered silent or cryptic and can be carried on plasmid or chromosomal DNA, and it does not confer corresponding phenotypic resistance to the associated antibiotic [9].

Gene silencing has been shown to result from point mutations (insertions, deletions, and substitutions) and larger genomic rearrangements, including deletions or insertions mediated by transposable elements. These genetic defects may disrupt gene expression by causing frameshifts, nonsense mutations, loss of translation initiation sequences, missense mutations in substrate-binding sites, separation of the promoter from the coding sequence, or truncation of regulatory genes that repress antibiotic resistance determinants [8,9]. Moreover, some genes may remain silent due to transcriptional or post-transcriptional regulatory mechanisms, including repressor overactivity, degenerated gene clusters, integron structure alterations, or xenogeneic silencing protein activity, even when the genetic sequence itself remains intact [10].

Some authors have also proposed distinguishing a related category termed “transiently silent acquired antimicrobial resistance” (tsaAMR), as SARM does not differentiate between isolates capable of reverting to resistance and those that cannot. The term tsaAMR, first proposed by Wagner et al., refers to acquired resistance genes with a corresponding phenotype that remains within the wild-type distribution or below the clinical breakpoint for susceptibility but which can revert or increase expression to a clinically relevant level of resistance upon genetic alteration [11].

*S. aureus* represents a major etiological factor in SSTIs. It exhibits exceptional virulence, including antimicrobial resistance, which highlights the pressing need to understand resistance dynamics in *S. aureus* infections. Here, silent genetic determinants can evade detection. Rigorous investigation of its resistome, i.e., phenotypic and genotypic resistance, is therefore imperative to address these critical clinical challenges.

This study aimed to compare phenotypic and genotypic resistance in *S. aureus* isolates from carriers and SSTIs and to elucidate *mupA* gene-silencing mechanisms in strains harboring the gene yet exhibiting susceptibility to mupirocin.

## 2. Results

### 2.1. Phenotypic Resistance to Selected Antibiotics

**Figure 1:**
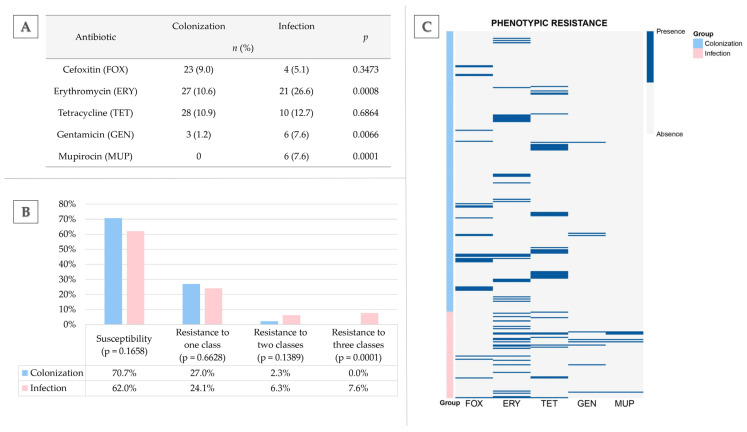
(**A**) Comparison of antibiotic resistance in *Staphylococcus aureus* strains from colonization (*n* = 256) and infection (*n* = 79); Fisher’s exact test; statistical significance set at *p* < 0.05; (**B**) the prevalence of resistance profiles in *S. aureus* isolates (one, two, or three antimicrobial classes); (**C**) heatmap of phenotypic resistance in *S. aureus* isolates from colonization (*n* = 256, blue) and skin/soft tissue infections (*n* = 79, pink). Bands indicate phenotypic resistance.

In total, 355 *S. aureus* isolates, including 256 isolated from the colonization of the upper respiratory tract and 79 from SSTIs in a clinical setting, were studied for their phenotypic resistance towards most clinically important antimicrobial classes, showing diversified patterns of phenotypic resistance (Figure 1C). Cefoxitin (FOX) was used as an MRSA marker indicating resistance to β-lactams (except for ceftaroline), erythromycin (ERY) as a marker of resistance to macrolides, tetracycline (TET) for tetracyclines, and gentamycin (GEN) for aminoglycosides. Mupirocin (MUP) was chosen due to its role in eradication protocols applied in *S. aureus* carriers.

In the entire *S. aureus* collection, the most prevalent was resistance to erythromycin (13.5%; 48/355) and tetracycline (10.7%; 38/355), followed by resistance to cefoxitin (7.6%; 27/355). The overall resistance to the two remaining antimicrobials (gentamicin and mupirocin) was generally low and equal to 2.5% (9/355) and 1.7% (6/355), respectively.

The analysis showed statistical differences in antimicrobial resistance in *S. aureus* isolates from colonization and infection. Resistance to erythromycin, gentamicin, and mupirocin was higher in clinical isolates than in those isolated from carriers (Figure 1A). Similar resistance rates in these two groups were noted towards cefoxitin (9% vs. 5.1%) and tetracycline (10.9% vs. 12.7%).

Despite relatively high resistance rates towards certain antimicrobials in both studied *S. aureus* groups, most isolates remained susceptible (70.7% in the colonization group and 62% in the infection group) or resistant to only one tested antimicrobial (27% in the colonization group and 24.1% in the infection group) (Figure 1B). The simultaneous resistance to two antimicrobials was present only in 2.3% of *S. aureus* strains from colonization (with four phenotypes FOX + ERY, one FOX + GEN, and one TET + GEN) and in 6.3% from those from infection (two phenotypes GEN + MUP, one FOX + TET, one FOX + ERY, and one ERY + GEN). The presence of multidrug resistance, i.e., resistance to at least one agent in three or more antimicrobial categories, was detected in six *S. aureus* isolates, all from clinical settings (*p* = 0.0001), which included the following phenotypes: ERY + GEN + MUP (two isolates), ERY + TET + MUP (two isolates), FOX + ERY + TET (one isolate), and ERY + TET + GEN (one isolate). Notably, phenotypic resistance to mupirocin was observed only in clinical isolates exhibiting co-resistance to other antimicrobial classes.

### 2.2. Prevalence of Antibiotic Resistance Genes

**Figure 2:**
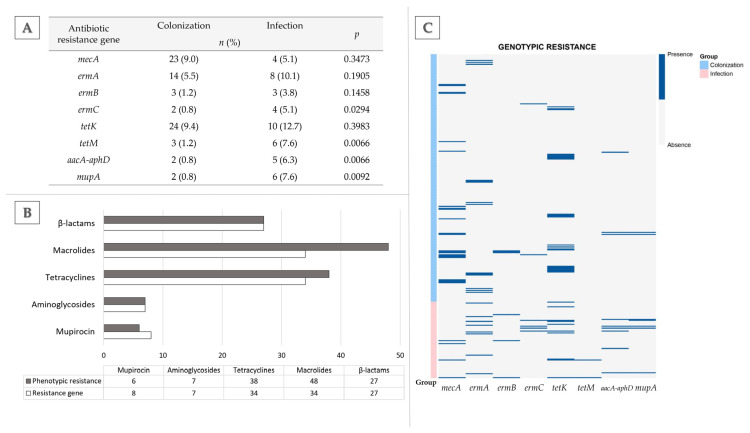
(**A**) Comparison of antibiotic resistance gene prevalence in *Staphylococcus aureus* isolated from colonization (*n* = 256) and infection (*n* = 79); Fisher’s exact test; statistical significance set at *p* < 0.05. (**B**) Concordance between the number of phenotypically resistant *S. aureus* isolates and the presence of ≥1 corresponding antibiotic resistance gene. (**C**) Heatmap of antimicrobial resistance gene (ARG) prevalence in *S. aureus* isolates from colonization (*n* = 256, blue) and skin/soft tissue infections (*n* = 79, pink). Bands indicate ARG presence (as detected by PCR).

The presence of several antimicrobial resistance genes (ARGs), most often underlying phenotypic resistance to the studied antimicrobial classes, was characterized, and their distribution in the studied *S. aureus* collection was visualized as a heatmap (Figure 2C). Responsible for methicillin resistance, *mecC* was the only gene not detected, while the *mecA* gene was the only one discerned in all MRSA isolates corresponding with this particular phenotypic mechanism of resistance.

What is interesting is that, in comparison with *S. aureus* isolates from the colonization group, isolates from the infection group showed a significantly higher prevalence rate of *ermC* (*p* = 0.0294), but not the two remaining genes conferring resistance to macrolides—*ermA* and *ermB*. This, in connection with statistically higher phenotypic resistance to erythromycin in isolates from infection, emphasizes the importance that *ermC* has in developing macrolide resistance in clinical settings. Similar observations were made regarding the prevalence of genes responsible for resistance to tetracyclines. Statistical analysis demonstrated that *tetM*, in opposition to *tetK* with comparable prevalence in both groups, was detected more often in isolates from the clinical setting than those from colonization (*p* = 0.0066).

The analysis of concordance between the number of phenotypically resistant *S. aureus* isolates and the presence of ≥1 corresponding antibiotic resistance gene showed that, for some phenotypically resistant isolates, ARGs were not detected (Figure 2B). This situation was evident for isolates resistant to tetracyclines and macrolides, where ARGs were not detected in 4 and 14 isolates, respectively. This outcome could be explained by the occurrence of less-studied ARGs not included in this work.

The analysis also revealed the possible presence of two nonfunctional resistance genes (*mupA*) carried by *S. aureus* isolates from the colonization group, the presence of which did not correspond to phenotypic resistance to mupirocin. One of the isolates (no. 187) was classified as MRSA resistant to aminoglycosides, while the other (no. 185) was resistant only to aminoglycosides, but both were proven to be related (based on the previous fingerprinting assays) and belonged to ST6295/CC8, which was investigated in the previous study [12]. After sequencing, the analysis revealed that the nonfunctional *mupA* gene in those isolates had an identical nucleotide sequence; thus, in a further part of this manuscript, only the sequence analysis for one isolate is presented.

### 2.3. Characterization of Silenced mupA Gene

**Figure 3:**
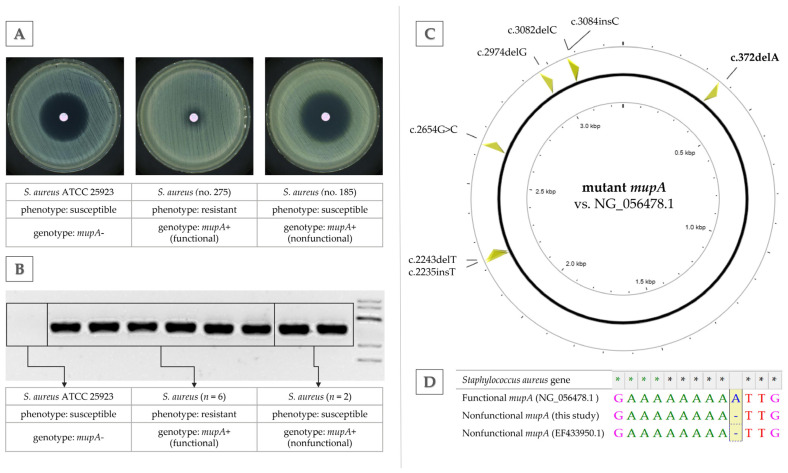
(**A**) Phenotypes of resistance to mupirocin in *Staphylococcus aureus* based on the disc-diffusion method with a mupirocin disc (200 µg) for representative isolates. (**B**) Genotypes of resistance to mupirocin in *S. aureus* based on *mupA* gene detection in PCR (size = 126 bp) for all isolates with high levels of mupirocin resistance and with *mupA* silencing. (**C**) Graphical representation of the *mupA* gene (mutant, nonfunctional, this study, PX906688) in comparison to the functional reference gene (NG_056478.1) with marked mutations in the nucleotide sequence. (**D**) Nucleotide alignment showing adenine deletion, causing a frameshift mutation and premature truncation.

Phenotypes and genotypes of resistance to mupirocin in *S. aureus* isolates carrying the nonfunctional *mupA* gene are presented in Figure 3A,B. Despite harboring the *mupA* gene, their phenotypes were identical to that of the reference strain *S. aureus* ATCC 25923, which showed full susceptibility (visible as a large inhibition zone in the disc-diffusion method). In contrast, all remaining six isolates harboring the *mupA* gene exhibited full phenotypic resistance with no inhibition zone (high-level of resistance), testifying that the gene was functional and expressed.

A complete sequence of the nonfunctional *mupA* gene (deposited in GeneBank under the accession number PX906688) was compared with available sequences in the National Center for Biotechnology Information (NCBI) database—with functional (GeneBank NG_056478.1) and nonfunctional *mupA* genes described by other authors (GeneBank EF433950.1). The reference functional *mupA* gene was 3275 bp long, encoding a 1024-amino-acid isoleucyl-tRNA synthetase (IleRS). In contrast, the nonfunctional *mupA* sequence from this study was 3264 bp long, with 99.15% nucleotide identity to the reference. All mutations detected in the gene in relation to the functional gene were marked (Figure 3C).

A critical frameshift mutation at position 372 (c.372 delA) within a poly(A) tract (a homopolymeric stretch of adenine residues prone to polymerase slippage and instability) introduced a premature stop codon, resulting in a truncated IleRS protein of only 887 amino acids. This made it substantially shorter than the full-length 1024-amino-acid enzyme essential for mupirocin resistance. Additional indels and substitutions relative to functional reference *mupA* included c.2243 delT, c.2235 insT, c.2654 G>C (missense), c.2974 delG, c.3082 delC, and c.3084 insC, further disrupting the coding sequence. These mutations, particularly the initial adenine deletion at position 372 causing the frameshift, rendered the *mupA* gene nonfunctional, similarly as observed by other authors (EF433950.1; Figure 3D), hence eliminating high-level mupirocin resistance despite PCR detection of the gene.

The expression of *mupA* across gene-harboring strains showed high biological variation (log_2_ FC ranging from −2.78 to 3.34) (Table 1). Two *S. aureus* isolates with a silenced *mupA* gene (“mutant” group) showed 2.97–10.12-fold upregulation, despite the presence of a nonsense mutation, truncating the protein. Among six isolates with functional genes (“normal” group), *mupA* expression spanned a 27-fold range (0.15–4.07×; log_2_ FC = −2.94 to +2.03), reflecting substantial biological heterogeneity. Mutant strains trended toward higher *mupA* expression than did normal, but statistical analysis revealed no statistical significance (t = −1.95, df = 3.1, *p* = 0.1139) due to high inter-strain variation and limited mutant number (*n* = 2). The analysis demonstrated unexpected upregulation of defective *mupA* in mutants alongside its high variation in normal strains, suggesting complex post-transcriptional or strain-specific regulation beyond simple genotypic predictions.

## 3. Discussion

In this study, we characterized phenotypic and genotypic antimicrobial resistance profiles in *S. aureus* isolates from upper respiratory tract colonization and SSTI while elucidating mechanisms of *mupA* gene silencing responsible for discrepancies between genotypic presence and phenotypic mupirocin susceptibility. These objectives enabled surveillance of resistance patterns and revealed frameshift mutations preventing functional gene expression. We demonstrated that phenotypic resistance among 355 *S. aureus* isolates was most prevalent with respect to erythromycin (13.5%; 48/355) and tetracycline (10.7%; 38/355), followed by cefoxitin (MRSA prevalence; 7.6%; 27/355), with lower rates for gentamicin (2.5%; 9/355) and mupirocin (1.7%; 6/355). Notably, resistance to erythromycin, gentamicin, and mupirocin was significantly higher in clinical isolates from SSTIs than in those from carriers. This outcome underscores distinct selective pressures between infection and colonization. Finally, MDR strains occurred more often in SSTIs than in carriers (*p* = 0.0001).

Antibiotic resistance is generally higher in bacteria from clinical settings due to selective pressures from therapeutic antibiotic use, host immune responses, and biofilm formation—factors less prominent in colonization [13,14]. For *S. aureus* SSTIs, which often represent endogenous infections arising from colonizing strains, this pattern reflects enrichment of resistant subpopulations during pathogenesis. However, *S. aureus* dynamics are distinctive, as colonizers serve as reservoirs while infection isolates exhibit amplified virulence and resistance profiles [15].

We demonstrated that, compared to *S. aureus* isolates from colonization, those from infection exhibited a significantly higher prevalence of *ermC* (*p* = 0.0294), but not the other macrolide resistance genes: *ermA* (*p* = 0.1905) or *ermB* (*p* = 0.1458). This pattern, alongside higher phenotypic erythromycin resistance in infection isolates, underscores the key role of *ermC* in clinical macrolide resistance in methicillin-susceptible *S. aureus* (MSSA). Recent findings confirm this—*ermC* predominates in erythromycin-resistant MSSA, causing bloodstream infections (up to 77% post—2013) that correlate with macrolide consumption [16].

In this study, phenotypic resistance to mupirocin was significantly higher among clinical *S. aureus* isolates from SSTIs (*p* = 0.001), whereas silent *mupA* resistance genes were detected exclusively in colonization isolates. Mupirocin (also known as pseudomonic acid A), first isolated from *Pseudomonas fluorescens* NCIB 10586, is used as a topical antibiotic for treating staphylococcal skin and wound infections and, more importantly, as the agent of choice for eradicating *S. aureus* or MRSA nasal carriage. Its crucial role in decolonization not only significantly reduces the risk of endogenous infections after multiple surgical procedures but also helps prevent MRSA spread in clinical settings [17,18].

The potent antistaphylococcal activity of Mupirocin relies on competitive inhibition of isoleucyl-tRNA synthetase, a native bacterial enzyme encoded by the *ileS* gene. *S. aureus* can exhibit high- or low-level resistance to mupirocin, with highly variable resistance rates worldwide that can undermine its effectiveness. High-level mupirocin resistance, characterized by minimum inhibitory concentration (MIC) ≥512 μg/mL, is conferred by the *mupA* gene (less often *mupB*), typically located on plasmids (rarely the chromosome), which encodes a structurally modified isoleucyl-tRNA synthetase with reduced drug-binding affinity [19,20]. Low-level resistance (MIC 8–256 μg/mL) arises from chromosomal mutations in *ileS*. Unlike high-level resistance, it is more prevalent in clinical settings and often leads to failure of nasal MRSA decolonization protocols [18,19,20].

Both high- and low-level mupirocin resistance facilitate clonal transmission, most likely through plasmid transfer via conjugation, including nonclinical settings such as long-term care facilities [18,21].

We determined the prevalence of silent *mupA* resistance genes in *S. aureus* to be 0.6% (2/355). Beyond our work, few other authors have directly investigated the frequency of their occurrence, with most studies focusing on silencing of penicillin or β-lactam resistance genes, as reported for *mecA* in *S. aureus* [22].

In one large-scale study of 1470 *S. aureus* isolates, the authors reported a frequency of SARM to be 3.1% (46/1470); however, the panel of genes investigated was broader than in our study [8]. Silent ARGs were identified for aminoglycosides (*aacA-aphD* and *ant4*), β-lactams (*mecA* and *blaZ*), clindamycin (*vga(A)v*), erythromycin (*ermA*), mupirocin (*mupA*), quinupristin-dalfopristin (*vga(A)v,* together with *ermA* or *ermC*), and tetracyclines (*tetK* and *tetM*). Notably, the prevalence of silent resistance towards mupirocin was the highest among all investigated antistaphylococcal drugs (0.75%; 11/1470) and comparable to our findings (0.6%; 2/335). The silencing mechanism was identical to that in our two characterized strains: a single-nucleotide deletion in the poly(A) tract. Of these eleven strains, nine were isolated from Israel (ST5/CC5), one from Ireland (ST22/CC22), and one from Greece (ST45/CC45) [8].

Kime et al. [8] also demonstrated that a single-nucleotide deletion in the poly(A) tract silences other ARGs, such as *tetM*, *blaZ*/*blaRI*, and *aad9*. Despite the limited number of studies on the prevalence of silent ARGs and their silencing mechanisms, this specific poly(A) tract mutation has also been reported elsewhere [23,24].

Homopolymeric tracts, such as poly(A) tract, are prone to mutations arising from slipped-strand mispairing during DNA replication. This process causes insertions or deletions (indels) that result in frameshifts, producing truncated or nonfunctional proteins. Conversely, this phenomenon enables rapid phenotypic diversification in bacteria, facilitating genome rearrangement by reactivating nonfunctional genes as needed for environmental adaptation [25,26].

Silent genes appear to be more prevalent in Gram-negative bacteria, though overall reports remain limited, making definitive comparisons challenging. Their rare detection likely reflects under-investigation rather than low prevalence rates [9]. Their presence has been described among the WHO’s priority pathogens, such as *Klebsiella pneumoniae*, *Escherichia coli*, *Salmonella* spp., and *Acinetobacter baumannii* [10]. These studies document various Gram-negative resistance genes rendered nonfunctional by mutations [27,28,29,30,31,32].

This phenomenon is also described in Gram-positive bacteria. Reports are primarily limited to *S. aureus* and *Enterococcus* spp. [33], but they have also been documented in *S. pneumoniae*, *Bacillus subtilis,* and *Clostridioides difficile* [34,35,36,37]. Due to their rare reporting and under-investigation (stemming from limitations in current diagnostic and experimental paradigms), the role of silent ARGs in shaping the epidemiological landscape of antimicrobial resistance remains largely unknown. The authors suggest that they act as hidden reservoirs of resistance, spreading silently, particularly in clinical settings. Moreover, studies have shown that ARG silencing can occur in vivo for the first time, often during infection treatment, making detection impossible with routine phenotypic diagnostics [9]. This phenomenon has even been documented in *mecA*-positive MSSA within a single patient during antibiotic therapy [24].

In other words, silent ARGs not only spread through horizontal gene transfer but can also reactivate upon transfer to a new host [10,38]. Supporting this, authors of a large-scale study on 1470 *S. aureus* strains assessed their reversion potential and found that approximately 96% regained phenotypic resistance [8]. Reversion frequencies (≥10^−9^ across various ARGs in *S. aureus*, >10^−6^ for *mupA*) enable bacteria to restore resistance during infection [8,23]. For poly(A) tract silencing specifically, reversion typically involves direct correction of the original mutation, with rates proportional to tract length (longer tracts revert more readily) [8]. This has been documented specifically for *mupA*, where adenine reinsertion restored high-level mupirocin resistance [23].

We demonstrated that all mupirocin-resistant SSTI *S. aureus* isolates exhibited co-resistance to ≥2 other classes (primarily erythromycin/gentamicin), unlike carrier strains. This MDR linkage, likely involving conjugative plasmids, underscores clinical selective pressures enriching *mupA* expression during pathogenesis [13]. Moreover, our study demonstrated high biological variability in *mupA* expression, regardless of whether strains harbored a defective or functional gene; notably, expression did not depend on the mutation itself but rather on other regulatory factors, and only a frameshift mutation prevented the occurrence of a resistant phenotype. This, combined with the high probability and documented cases of phenotype reversal from susceptible to resistant during infection, delivers a compelling take-home message on mupirocin resistance dynamics.

While this study provides insights into *mupA* silencing and resistome profiling in *S. aureus*, several limitations should be acknowledged. Only two isolates with silenced *mupA* were identified (0.6% prevalence; both from colonization). While this rarity mirrors previous reports, the absence of infection-derived silenced isolates and the small sample size preclude broad clinical extrapolations. Additionally, our study did not assess mutation rates, as prior works have demonstrated analogous *mupA* activation. Moreover, RT-qPCR upregulation lacks protein-level validation (e.g., Western blot) due to antibody unavailability; future studies should confirm truncated *mupA* expression. Finally, phenotypically resistant but PCR-negative isolates likely harbor untargeted ARGs or efflux mechanisms; future work should expand ARG profiling for comprehensive characterization.

## 4. Materials and Methods

### 4.1. Collection of Isolates

The studied material consisted of 344 *S. aureus* isolates: 265 obtained from colonized individuals from the upper respiratory tract (nasal vestibules or throat) and 79 clinical strains derived from SSTIs. Isolates from carriers were collected in 2018 and 2019, whereas clinical isolates were collected in 2021 and 2022. All samples originated from the same geographic region—the city of Lublin, located in eastern Poland. The isolate collection was stored at −70 °C in a mixture of Tryptic Soy Broth (Biomaxima, Lublin, Poland) and glycerol (Polchem, Gliwice, Poland) at a 1:1 ratio in the Department of Pharmaceutical Microbiology, Medical University of Lublin, Poland.

### 4.2. Antimicrobial Susceptibility Testing

Determination of antimicrobial resistance profile was conducted and interpreted according to the European Committee on Antimicrobial Susceptibility Testing (EUCAST, V. 13.0). The following antimicrobials were used in the disc-diffusion assay: cefoxitin (30 µg), erythromycin (15 µg), and mupirocin (200 µg). In contrast, resistance to tetracycline and gentamicin was determined using the Vitek 2 Compact and AST-P644 cards (Biomerierux, Marcy-l’Étoile, France). Antibiotic discs were purchased from Becton Dickinson (Franklin Lakes, NJ, USA). This panel of antimicrobials was chosen to reflect *S. aureus* resistance to major antimicrobial classes—β-lactams, macrolides, aminoglycosides, and tetracyclines. Mupirocin was chosen because of its role in the eradication of *S. aureus* colonization. MRSA was detected using the cefoxitin-screen test according to the previously mentioned EUCAST recommendations. The results were presented as heatmaps, constructed by way of the pheatmap package [39] in R software (version 4.4.1) [40]. Statistical comparisons of phenotypic antimicrobial resistance between colonization and infection isolates were performed via Fisher’s exact test, with statistical significance set at *p* < 0.05 (GraphPad Prism version 6.0; GraphPad Software, San Diego, CA, USA).

### 4.3. Detection of Selected Antimicrobial Resistance Genes Using PCR

Genetic material was isolated using the spin-column method according to the protocol provided by the manufacturer (Genomic Mini AX Bacteria, A&A Biotechnology, Gdańsk, Poland), as described in detail previously [41].

Selected ARGs conferring phenotypic resistance were detected: *mecA*, *mecC* (resistance to β-lactams, genetic marker of MRSA), *ermA*, *ermB*, *ermC* (resistance to macrolides), *tetK*, *tetM* (resistance to tetracyclines), *aacA-aphD* (resistance to aminoglycosides), and *mupA* (resistance to mupirocin). Details on the primer sequences, PCR conditions, and amplicon sizes were described previously: *mecA* [42], *mecC* [43], *ermA* and *ermC* [44], *ermB* [45], *tetK*, *tetM*, *aacA-aphD* [46], and *mupA* [47]. Electrophoresis was conducted under 120 V by employing standard agarose gel (1.5% of agarose; EURx, Gdańsk, Poland) stained with SimplySafe dye (EURx, Gdańsk, Poland) and a molecular marker of appropriate weight (EURx, Gdańsk, Poland). Several reference *S. aureus* strains from the American Type Culture Collection (ATCC) and the Collection of the Department of Pharmaceutical Microbiology (DPM) were used as controls: *S. aureus* ATCC 29213, *S. aureus* ATCC 43300, *S. aureus* ATCC BAA −1707, DPM −13, DPM −137. The results were presented as heatmaps, constructed utilizing the pheatmap package [39] in R software (version 4.4.1) [40]. Statistical comparisons of ARGs prevalence between colonization and infection isolates were performed via Fisher’s exact test, with statistical significance set at *p* < 0.05 (GraphPad Prism version 6.0; GraphPad Software, San Diego, CA, USA).

### 4.4. Identification and Sequencing of Silenced Antimicrobial Resistance Genes

The results of phenotypic and genotypic antimicrobial resistance were compared to identify disconcordance, i.e., the presence of silent ARGs, where the gene was detected by PCR but the isolate remained susceptible to the tested antimicrobial.

The only silenced gene detected—the *mupA* gene—was sequenced with Sanger sequencing, as described previously, using six pairs of primers [23]. The obtained sequences were aligned in MEGA11 [48] to determine the complete *mupA* gene sequence (length 3233 bp), and they were deposited in the NCBI database (receiving accession number PX906688). The sequence was then compared with other *mupA* sequences available in the NCBI database. These included an NG_056478.1 sequence (representing functional *mupA* in a *S. aureus* isolate exhibiting a high-level of mupirocin resistance) and an EF433950.1 sequence (present in an *S. aureus* isolate with previously documented nonfunctional (silenced) *mupA*). The complete *mupA* gene sequence was maintained on a plasmid construct, with all mutations and polymorphisms (mutations being identified relative to the reference gene NG_056478.1) presented in the assembled contig, using VectorBuilder [49]. Additionally, the analyzed *mupA* sequence was translated into amino acid sequences, employing NCBI ORF Finder [50] to determine whether the identified point mutations resulted in the shifting of an open reading frame for gene translation.

### 4.5. Reverse Transcription Qualitative PCR (RT-qPCR) Analysis of mupA Gene Expression

Eight *S. aureus* strains harboring the *mupA* gene were analyzed: two with phenotypic mupirocin susceptibility (no 185 and 187), designated as the “mutant” group, and six exhibiting high-level mupirocin resistance (no 275, 276, 277, 282, 284, and 330), classified as the “normal” group. Strains were grown in BHI broth (Oxoid, Thermo Scientific, Basingstoke, UK) at 37 °C for 24 h prior to RNA extraction. After incubation, the bacterial suspension was adjusted to 10^6^ jtk/mL. Total RNA was isolated with TRIzol reagent (Invitrogen, Carlsbad, CA, USA), following the manufacturer’s instructions. Its concentration and quality were measured through a NanoDrop spectrophotometer (Thermo Scientific, Wilmington, DE, USA). RNA was stored at −20 °C until analysis.

Primers for the *mupA* gene were designed and validated for specificity to *S. aureus* by employing Primer-BLAST (NCBI) [51] (amplicon size: 126 bp; Tm: ~60 °C). Sequences were as follows: forward 5′-CCAACTGCAAATGGCCTTCC-3′ and reverse 5′-GCCATGGGTATCCCATCCTG-3′. Reference genes *gmk* and *gyrA* were selected based on a previously published report [52].

RT-qPCR was performed in triplicate by means of an SG OneStep RT-qPCR Kit (EURx, Gdańsk, Poland) on a LightCycler 96 (Roche, Basel, Switzerland). Reactions (20 µL total volume) were prepared according to the protocol provided by the manufacturer, with no-template controls. Cycling conditions followed the manufacturer’s protocol as well: 30 min at 65 °C (reverse transcription), 20 s at 98 °C (initial denaturation), and 40 cycles of 10 s at 98 °C/20 s at 60 °C, followed by melting curve analysis (60–95 °C). Amplification specificity was confirmed by melting curves.

Relative *mupA* expression was quantified via the 2^−ΔΔCt^ method [53], normalizing to the geometric mean of *gmk*/*gyrA* against a calibrator (average ΔCq for strains with functional *mupA*). Differences were assessed by Welch’s unpaired t-test in GraphPad Prism (version 6.0; GraphPad Software, San Diego, CA, USA) with statistical significance set at *p* < 0.05. All experiments adhered to MIQE guidelines [54].

## 5. Conclusions

The emergence and spread of silent ARGs represent a serious yet under-investigated phenomenon with potentially profound clinical consequences. Our findings on *mupA* silencing in *S. aureus* isolates from carriers underscore that these silent reservoirs can potentially enhance phenotype shifts from susceptibility to resistance during endogenous infections, exacerbating treatment failures and transmission risks. This hidden dimension of antimicrobial resistance demands urgent attention and targeted therapeutic strategies that are essential to unravel its epidemiology, mechanisms, and clinical impact. Addressing silenced ARGs is not merely an academic pursuit, but it is a necessary step toward preserving mupirocin and other antibiotics in the face of the post-antibiotic era.

## Figures and Tables

**Table 1:** Reverse transcription qualitative PCR (RT-qPCR) analysis of *mupA* expression in *Staphylococcus aureus* isolates with a silenced gene (mutant) versus a functional gene (normal).

Isolate	Group	Cq *mupA*	Cq *gmk*	Cq *gyrA*	Cq ref	ΔCq	log_2_ FC (−ΔΔCq)	Fold Change
		AM ± SD			GM			
185	Mutant	21.33 ± 0.80	23.16 ± 0.20	25.44 ± 0.54	24.27	−2.94	3.34	10.12 × ↑
187		23.86 ± 0.53	23.51 ± 0.48	26.65 ± 0.37	25.03	−1.18	1.57	2.97 × ↑
275	Normal	29.99 ± 1.72	23.86 ± 0.34	30.14 ± 0.31	26.81	3.18	−2.78	0.15 × ↓
276		24.36 ± 0.20	23.85 ± 0.61	26.49 ± 0.12	25.14	−0.78	1.18	2.26 × ↑
277		24.57 ± 0.32	22.53 ± 0.61	24.01 ± 0.18	23.25	1.31	−0.92	0.53 × ↓
282		24.93 ± 0.32	23.56 ± 0.24	25.06 ± 0.62	24.30	0.63	−0.24	0.85 × ↓
284		23.46 ± 0.21	23.04 ± 0.36	24.37 ± 0.68	23.70	−0.24	0.64	1.55 × ↑
330		25.17 ± 0.07	24.26 ± 0.13	29.61 ± 0.43	26.80	−1.63	2.02	4.07 × ↑
Mutant		22.59 ± 0.19	23.34 ± 0.20	26.04 ± 0.11	24.65	−2.06	-	*p* = 0.1139 *
Normal		25.41 ± 0.62	23.52 ± 0.19	26.61 ± 0.23	25.02	0.40	-	

AM—Arithmetic mean; GM—geometric mean; SD—standard deviation; ΔCq = Cq *mupA* − Cq ref (*gmk*, *gyrA*); ΔΔCq = ΔCq *mupA* − ΔCq calibrator (normal group); ↑ — upregulation; ↓ — downregulation;* Welch’s unpaired *t*-test comparing log_2_ FC between mutant (*n* = 2) and wild-type (*n* = 6) strains.

## Data Availability

All datasets generated in this study are available in the RepOD repository: https://doi.org/10.18150/5RDKUM (accessed on 15 March 2025) [Kasela, M. Antimicrobial resistance and silenced genes in *Staphylococcus aureus*. RepOD 2025, V1].
